# Targeting the ERβ/HER Oncogenic Network in *KRAS* Mutant Lung Cancer Modulates the Tumor Microenvironment and Is Synergistic with Sequential Immunotherapy

**DOI:** 10.3390/ijms23010081

**Published:** 2021-12-22

**Authors:** Abdulaziz A. Almotlak, Mariya Farooqui, Adam C. Soloff, Jill M. Siegfried, Laura P. Stabile

**Affiliations:** 1 Department of Pharmacology, College of Clinical Pharmacy, Imam Abdulrahman Bin Faisal University, Dammam 34212, Saudi Arabia; aaalmotlak@iau.edu.sa; 2 Department of Pharmacology, Masonic Cancer Center, University of Minnesota, Minneapolis, MN 55455, USA; Mariya2020umn@gmail.com (M.F.); jsiegfri@umn.edu (J.M.S.); 3 Department of Cardiothoracic Surgery, UPMC Hillman Cancer Center, University of Pittsburgh School of Medicine, Pittsburgh, PA 15213, USA; adamsoloff@pitt.edu; 4 Department of Pharmacology and Chemical Biology, UPMC Hillman Cancer Center, University of Pittsburgh, Pittsburgh, PA 15213, USA

**Keywords:** NSCLC, immunotherapy, ER, TME, HER, anti-PD-1, PAM50, KRAS

## Abstract

High ERβ/HER oncogenic signaling defines lung tumors with an aggressive biology. We previously showed that combining the anti-estrogen fulvestrant with the pan-HER inhibitor dacomitinib reduced ER/HER crosstalk and produced synergistic anti-tumor effects in immunocompromised lung cancer models, including *KRAS* mutant adenocarcinoma. How this combination affects the tumor microenvironment (TME) is not known. We evaluated the effects of fulvestrant and dacomitinib on murine bone marrow-derived macrophages (BMDMs) and CD8+ T cells, and tested the efficacy of the combination in vivo, using the *KRAS* mutant syngeneic lung adenocarcinoma model, FVBW-17. While this combination synergistically inhibited proliferation of FVBW-17 cells, it had unwanted effects on immune cells, by reducing CD8+ T cell activity and phagocytosis in BMDMs and inducing PD-1. The effects were largely attributed to dacomitinib, which caused downregulation of Src family kinases and Syk in immune cells. In a subcutaneous flank model, the combination induced an inflamed TME with increased myeloid cells and CD8+ T cells and enhanced PD-1 expression in the splenic compartment. Concomitant administration of anti-PD-1 antibody with fulvestrant and dacomitinib was more efficacious than fulvestrant plus dacomitinib alone. Administering anti-PD-1 sequentially after fulvestrant plus dacomitinib was synergistic, with a two-fold greater tumor inhibitory effect compared to concomitant therapy, in both the flank model and in a lung metastasis model. Sequential triple therapy has potential for treating lung cancer that shows limited response to current therapies, such as *KRAS* mutant lung adenocarcinoma.

## 1. Introduction

Lung cancer is the leading cause of cancer-related mortality in the US [1]. Despite the advancements in understanding its biology, the 5-year survival rate is less than 20% [2]. Current treatment for advanced non-small cell lung cancer (NSCLC) relies on cytotoxic chemotherapy, targeted therapies, and immune-checkpoint blockade. However, intrinsic and acquired resistance are very common and lead to early relapse. Safe combinations and rationale sequencing of different anticancer drugs that target multiple aspects of tumorigenesis could enhance antitumor effects and improve patient outcomes.

The tumor microenvironment (TME) is a key determinant of lung tumor progression, and the reciprocal cross-talk between tumor cells and immune cells in the TME also plays a crucial role in controlling tumor fate. Tumor-associated macrophages (TAMs), which largely display an M2-phenotype, exhibit pro-tumorigenic functions and express CD206, programmed death-1 (PD-1) and its ligand (PD-L1), and IL-10 [3,4,5]. Additionally, T-lymphocytes play a major role in the antitumor immune response. T-lymphocyte functions are tightly controlled by the TME. The PD-1/PD-L1 inhibitory immune checkpoint pathway represents the major immune-escape mechanism for tumors, and agents that target this pathway have redefined the standard of care treatment of NSCLC. However, the overall response rate for PD-1/PD-L1 inhibitors, when used as a monotherapy, is less than 30% [6].

We previously showed that targeting estrogen receptor β (ERβ) with the ER blocker fulvestrant (F), together with targeting the family of epidermal growth factor receptors (HERs) with the pan-HER tyrosine kinase inhibitor (TKI) dacomitinib (D), had a synergistic antitumor effect in ERβ+ lung cancer, producing a gene signature that better predicts clinical outcomes [7]. In addition to blocking EGFR, blocking HER2 and HER3 signaling was important in achieving synergy with F+D [7]. These effects were observed in human lung tumor xenografts (*EGFR* WT, *EGFR* mutant, and *KRAS* mutant) propagated in immunocompromised mice, but the effects on the immunocompetent TME could not be elucidated. Evidence suggests that pan-HER TKIs have dual immunomodulatory effects. D inhibits Src kinases, including lymphocyte specific tyrosine kinase (LcK), while upregulating MHC complexes in cancer cells [8,9]. Furthermore, F was found to be one of the top drugs that sensitized NSCLC cells to immune-mediated lysis [10]. Thus, while F may promote anti-tumor immunity, D may reduce the activity of immune cells, suggesting that the antitumor effect of F+D could be further improved by adding an immune checkpoint blocker (ICB), to overcome the undesirable effects on immune cells mediated by D.

Here, we evaluated the immunomodulatory effects of F+D in vitro and in vivo using the novel syngeneic *KRAS+* murine lung cancer model, FVBW-17, derived from a lung adenocarcinoma induced by exposure of an FVB/N mouse to the tobacco carcinogen NNK [11]. *KRAS*+ lung cancer is known to be dependent on HER2/HER3 signaling [12,13] and often express ERβ [7,14], making it potentially responsive to a pan-HER inhibitor in combination with an ER blocker. In FVBW-17 cells, D produced an anti-tumor effect but also reduced macrophage phagocytosis, induced PD-1 expression, and inhibited spleen tyrosine kinase (Syk) activity and Src family kinases. The addition of F to D could not overcome these effects. Combining F+D with an anti-PD-1 antibody improved treatment response. Sequential triple therapy showed synergy in a subcutaneous (s.c.) model, which was confirmed in an intrapulmonary model, where sequential administration was superior for reducing growth of FVBW-17 cells seeded into the lungs. The ability of F+D to induce an inflamed TME and upregulate PD-1, both highly predictive factors for sensitivity to anti-PD-1 agents [15,16], makes F+D an attractive regimen to administer in *KRAS*+/ER+ lung cancer prior to an ICB. Use of an ICB after a TKI might also reduce the toxicities that have been observed in patients when an ICB was concomitantly given with targeted therapies [17].

## 2. Results

### 2.1. While Potently Inhibiting KRAS+ Lung Tumor Cell Growth, F+D Induced PD-1 Expression in Bone Marrow Derived Macrophages

**Figure 1:**
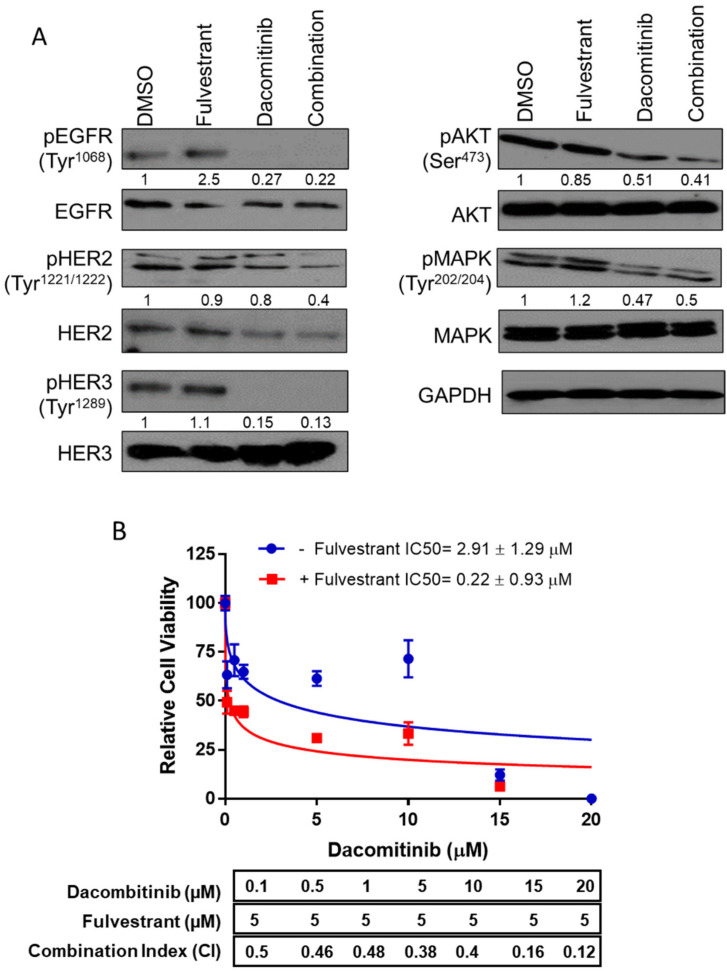
Effect of F+D on HER phosphorylation and cell proliferation in FVBW-17 cells. (**A**). FVBW-17 cells were treated with control (DMSO), fulvestrant (5 µM), dacomitinib (10 µM), or a combination for 24 h. Cell lysates were prepared and immunoblotted with the indicated antibodies. Quantitation is shown below each blot relative to control. (**B**). FVBW-17 cell viability was examined using the CellTiter 96 reagent after 72 h treatment with fulvestrant (5μM) combined with the indicated concentrations of dacomitinib. IC50 values for viability were calculated by nonlinear regression. Results are presented as mean ± SEM of six replicates. CI is shown beneath the graph for each condition. CI < 1 indicates synergy.

**Figure 2:**
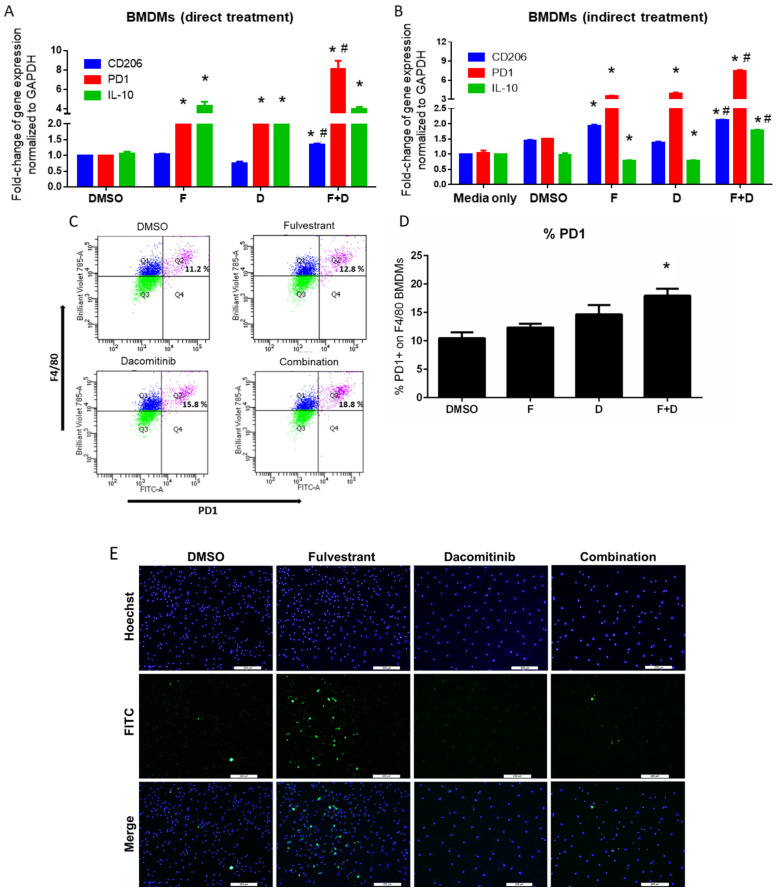

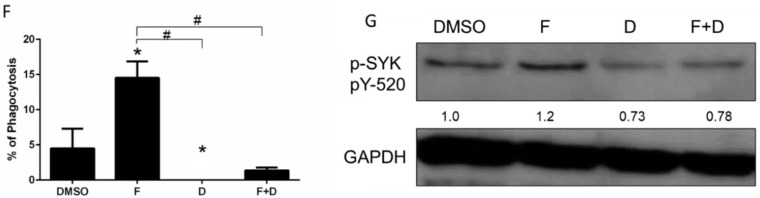
Effect of F+D on M2 related phenotypes and phagocytic activity of BMDMs in vitro. (**A**). Direct treatment of BMDMs. Resting state BMDMs, were treated for 6 h with F (5 µM), D (10 µM), or F+D, then media was changed and RNA was isolated using 18 h post-treatment (total time 24 h). *GAPDH* was used as a control and data were normalized relative to DMSO. Differences were assessed by two-tailed Student’s *t* test and considered significant at *p* ≤ 0.05: * compared to DMSO or # compared to single treatment groups. (**B**). Indirect treatment of BMDMs. Resting state BMDMs were treated for 24 h with FVBW-17 CM. FVBW-17 cells were first treated with F (5 µM), D (10 µM), or F+D for 6 h, after which the media were replaced with serum-free media for 18 h. The media was then collected and added to BMDMs for 24 h. *GAPDH* was used for normalization of each group, and data were then expressed relative to BMDM media only (not exposed to media from FVBW-17 cells). Differences were assessed by ANOVA followed by two-tailed Student’s *t* test of individual comparisons and considered significant at *p* ≤ 0.05: * compared to DMSO or # compared to single treatment groups. (**C**). Representative example of flow cytometry performed on BMDMs treated for 6 h with DMSO control (top left) fulvestrant (5 µM; top right), dacomitinib (10 µM, bottom left), or the combination (bottom right). Cells were double stained with cell surface markers for macrophages (F4/80) and PD-1. Quadrant 2 (Q2) represents the double positive F4/80+/PD-1+ population. (**D**). Quantitation of F4/80+/PD-1+ population by treatment group. Data are represented as mean ± SD. Differences were determined by ANOVA followed by two-tailed Student’s *t* test of individual comparisons and considered significant at *p* ≤ 0.05; * compared to DMSO. (**E**). Representative phagocytosis assay performed on BMDMs following treatment for 6 h with fulvestrant (5 µM), dacomitinib (10 µM), or the combination. Images were captured by microscopy at 10×. (Scale = 200 µM). (**F**). Quantification of phagocytic macrophages normalized to the total macrophages. Mean ± SD. Differences were considered significant at *p* ≤ 0.05 determined by ANOVA followed by two-tailed Student’s *t* test: * compared to DMSO or # compared to single treatments. (**G**). Effect of F+D treatment on expression of p-Syk. BMDMs were treated for 6 h with F (5 µM), D (10 µM), or F+D. Densitometric quantification was performed using GAPDH as a loading control.

To study the impact of F+D in an immunocompetent model, we utilized the syngeneic murine cancer cell line, FVBW-17 [11]. The FVBW-17 cell line is the only available murine adenocarcinoma cell line derived from exposure to a tobacco carcinogen, and carries an activating *KRAS^G12D^* mutation and a mutant *TP53* gene [11]; these cells also express EGFR, HER2, HER3, ERβ, and PD-L1 (Supplemental Figure S1). FVBW-17 cells represent a unique model that bears similarity to human tobacco-induced lung adenocarcinoma and can be propagated in syngeneic immunocompetent FVB mice [11]. D alone or F+D inhibited phosphorylation of EGFR, HER2, HER3, Akt, and MAPK1/2 kinases (Figure 1A). Additionally, F+D suppressed the FVBW-17 growth in vitro to a greater extent than D alone (Figure 1B). F alone showed limited growth suppression [7] and was used at a constant concentration of 5 µM. F+D had a combination index (CI) of 0.12–0.5, indicating strong synergy [18]; recapitulating effects seen previously in human NSCLC [7]. To assess F+D in macrophages, we utilized syngeneic bone marrow derived macrophages (BMDMs). F+D had little to no toxicity on resting macrophages (Supplemental Figure S2). *PD-1*, *CD206*, and *IL-10* expression were assessed in BMDMs treated directly with F+D (Figure 2A) or indirectly with conditioned media (CM) from FVBW-17 cells previously exposed to F+D. (Figure 2B). PD-1 is known to be inducible in M0 macrophages, for example with lipopolysaccharide [19]. In the direct treatment assay, F+D increased *PD-1* expression by eight-fold, compared to two-fold with single drugs. *CD206* mRNA was also increased by 50% with F+D compared to single drugs. *IL-10* gene expression was significantly induced in all groups. 

In BMDMs treated with FVBW-17 CM (indirect treatment assay), FVBW-17 cells were treated as above, but after 6 h, the media was replaced with serum-free media. The serum-free CM was collected 18 h later, then added to M0 macrophages. CM from F+D treated tumor cells increased *PD-1* mRNA of BMDMs by seven-fold compared to three-fold with single drugs. *IL-10* mRNA increased by 50%, and *CD206* mRNA was induced two-fold with F+D compared to control (Figure 2B). *PD-1* upregulation was likely due, in part, to the ability of dacomitinib to inhibit the cytokine production of TGFβ (66% reduction compared to control), as measured by a cytometric bead array in the CM after treatment (data not shown). Other cytokines (CCL2, IFNγ, IL-12, IL-10, TNFα, IL-4, and IL-1β) evaluated were not significantly changed, suggesting that additional unidentified tumor-derived soluble factors promote alternative activation in BMDMs. This shows that in addition to direct effects on M0 macrophages, F+D maximally modulates tumor–macrophage interactions to alter the phenotype of macrophages. F+D also increased PD-1 protein expression in BMDMs when analyzed by flow cytometry (Figure 2C). The proportion of PD-1+ macrophages was significantly elevated by F+D compared to DMSO (*p* = 0.01), while single treatments increased PD-1 positivity but the effect was not significant (Figure 2D).

The phagocytic activity of BMDMs was also modulated by F+D. Using FITC-conjugated IgG latex beads, both D and F+D impaired the phagocytic activity of macrophages (Figure 2E). F demonstrated three-fold enhanced engulfment, consistent with the known ability of estrogen to reduce macrophage function [20], while D severely suppressed the phagocytosis process. With F+D, the phagocytic function was slightly enhanced compared to D alone, but the induction seen with F was repressed (Figure 2F). Mechanistically, we found that D alone or in combination with F significantly reduced p-Syk (Tyr520) (Figure 2G), an essential kinase in macrophage signaling [21]. Both Syk and Src kinases communicate with each other to orchestrate intracellular signaling, and inhibiting Src will potentially affect Syk activity [22]. These data suggest that while F+D treatment strongly suppresses the growth of cancer cells, D may have unwanted effects on macrophages, by suppressing kinases essential for macrophage function, which cannot be overcome by F.

### 2.2. F+D Inhibited CD8+ T Cell Activity and Induced PD-1 

**Figure 3:**
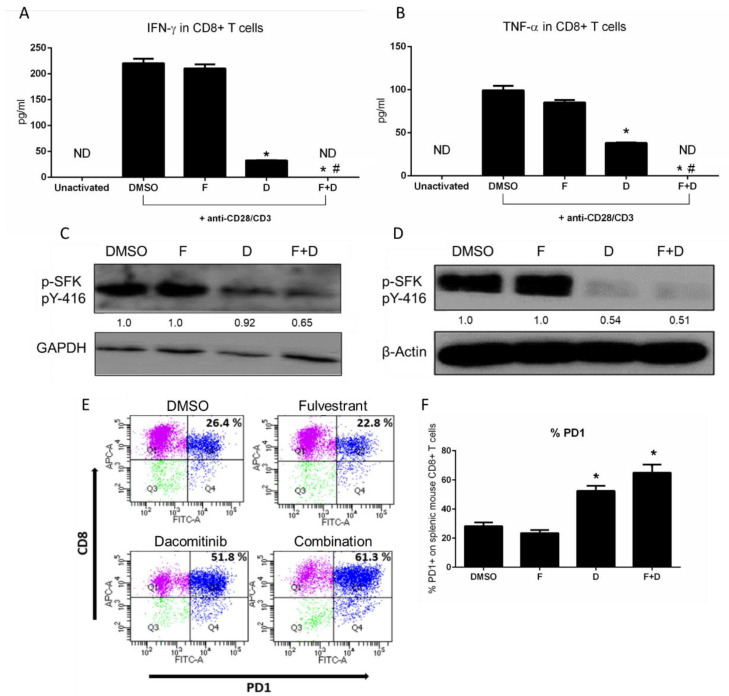
Effect of F+D treatment on mouse CD8+ T cell cytokine release and PD-1 positivity. (**A**). IFN-ϒ and (**B**). TNF-α release from mouse CD8+ T cells. T cells isolated from mouse spleens were activated with anti-CD28/CD3 before treatment with F (5 µM), D (10 µM), or F+D for 6 h. Media were collected for ELISA. Statistical differences were evaluated using the two-tailed Student’s *t* test and considered significant at *p* ≤ 0.05: * indicates significance compared to DMSO or # compared to single treatments. (**C**). Effect of F+D on expression of P-SFK (Tyr 416) in mouse CD8+ T cells. CD8+ T cells were isolated and activated with anti-CD28/CD3 for 24 h, then treated for 6 h with F (5 µM), D (10 µM), or F+D. Densitometric quantification was performed using GAPDH as a loading control. (**D**). Effect of treatments on P-SFK (Tyr 416) in Jurkat human T cells and β-actin as a loading control. (**E**). Representative flow cytometric analysis of PD-1 positivity on mouse CD8+ T cells following treatment. CD8+ T cells were stimulated with anti-CD28/CD3 for 24 h then treated for 24 h with fulvestrant (5 µM), dacomitinib (10 µM), or a combination. Quadrant 2 represents the CD8+/PD-1+) population. (**F**). Quantitation of PD-1 positivity on CD8+ T cells. Data are represented as the mean ± SD. Statistical differences were evaluated using ANOVA followed by a two-tailed Student’s *t* test. * *p* ≤ 0.05 compared to DMSO and F.

We then investigated the immunomodulatory effects of F+D treatment on T cells. In primary stimulated CD8+ murine T cells, F+D completely suppressed the secretion of IFN-ϒ and TNF-α after 6 h, while F slightly reduced TNF-α and had no effect on IFN-ϒ release. D significantly reduced both IFN-ϒ and TNF-α (Figure 3A,B). D, especially in combination with F, reduced the phosphorylation of Src (Tyr416), which is located in the activation loop of Src family kinases, while F alone had no effect (Figure 3C). The same effect was observed in Jurkat human T cells (Figure 3D).

PD-1 protein expression was upregulated following a 6 h F+D treatment. As in macrophages, PD-1 was induced by 60% compared to 50% in D, 22.8% in F, and 26.5% in the DMSO group, and this effect was mainly driven by D (Figure 3E,F). Together, these data suggest that F+D impairs T cell activity, an effect that is largely mediated by D. The increased expression of PD-1 on both macrophages and CD8+ T cells induced by F+D provides a rationale for adding an anti-PD-1 agent to improve the net antitumor effect and potentially overcome the adverse effects of F+D on the TME.

### 2.3. Triple Therapy Increases CD8+ T Lymphocytes and Produces an Inflamed TME

To assess the effects of adding anti-PD-1 antibody to F+D in vivo, FVB/N mice were engrafted subcutaneously with FVBW-17 cells. One week after injection, mice were randomized to receive F, D, anti-PD-1 antibody, F+D, F+anti-PD-1, D+anti-PD-1, or triple therapy for one week. The anti-PD-1 antibody used had previously shown efficacy in lung cancer [6]. We assessed the immediate effects of these agents on the TME and on the spleens of tumor-bearing mice, as the spleen is one of the major reservoirs of lymphocytic and inflammatory cells, and can be modulated by flank tumors [23]. Tumors and spleens from three tumor-bearing mice per group were pooled for flow cytometry, to examine T cells and macrophages and obtain sufficient events for evaluation (see example gating strategy in Supplemental Figure S3).

**Figure 4:**
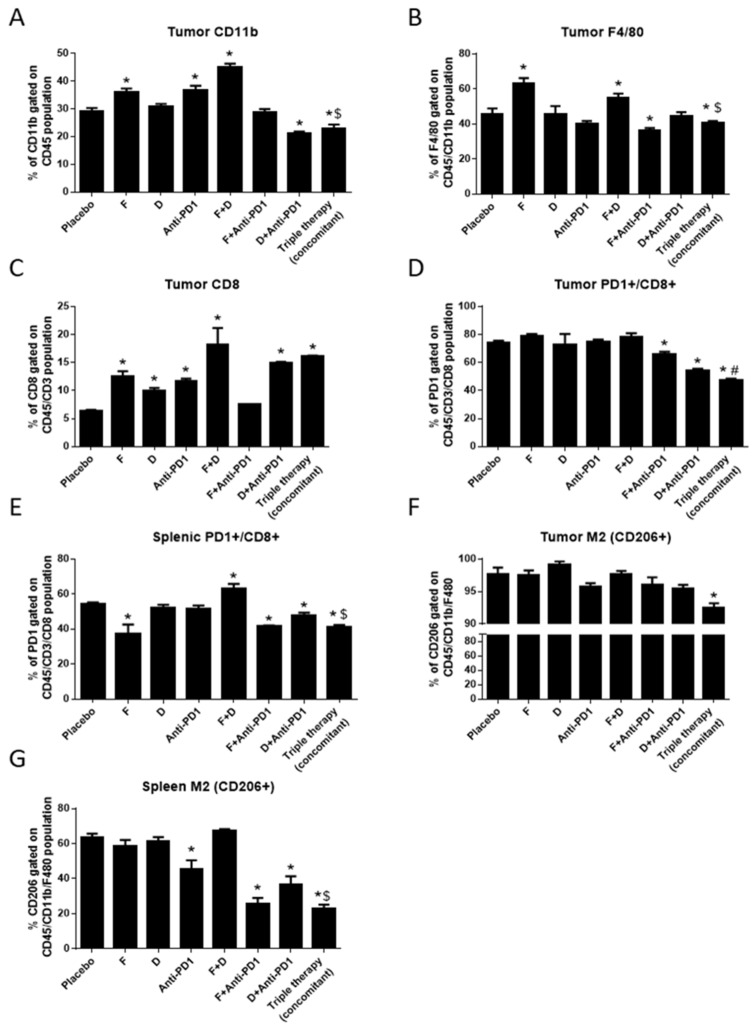
Triple therapy effect on T cell and M2 macrophage infiltration in the TME. FVBW-17 cells were s.c. transplanted into the flanks of FVBN/mice and treated with placebo, F, D, anti-PD-1, or dual and triple therapy for one week. Tumors and spleens were harvested and single cell suspensions prepared for flow cytometry. Percentage of the tumor cell population that is (**A**). CD11b+/CD45+; (**B**). F4/80+/CD45+/CD11b+; (**C**). CD8+/CD45+/CD3+; (**D**). CD45+/CD3+/CD8+/PD-1+. (**E**). Percentage of the splenic population that is CD45+/CD3+/CD8+/PD-1+. (**F**). Percentage of the tumor cell population that is F4/80+/CD45+/CD11b+/CD206+. (**G**). Percentage of the splenic cell population that is F4/80+/CD45+/CD11b+/CD206+. Data were analyzed by ANOVA, followed by *t*-test and considered significant at *p* ≤ 0.05: * indicates significance compared to placebo, $ is significant compared to F+D, and # is significant compared to all other treatment groups. Data are represented as mean ± SEM.

F+D significantly increased the infiltration of CD11b+ myeloid cells into tumors compared to placebo, F, or D alone, while triple therapy significantly reduced these cells compared to F+D (Figure 4A). The percentage of F4/80 macrophages was significantly higher in F and F+D groups, while D or anti-PD-1 antibody alone had no effect (Figure 4B). Triple therapy also significantly reduced the macrophage infiltration induced by F+D and was also lower than the placebo (Figure 4B). Importantly, the CD8+ T cell population showed a three-fold increase in F+D, D+anti-PD-1, or the triple therapy, and a two-fold increase in F, D, and anti-PD-1, when given alone compared to placebo. These data suggest that F+D induced an inflamed TME, by increasing the proportion of myeloid and CD8+ T cells found in the TME, effects that could potentiate the efficacy of PD-1 inhibition.

The proportion of PD-1+/CD8+ T cells in the tumor compartment was significantly lower in F+anti-PD-1 and D+anti-PD-1 and the triple therapy group compared to placebo (Figure 4D). Triple therapy was significantly lower than the D+anti-PD-1 (*p* = 0.03) or F+anti-PD-1 group (*p* = 0.0044). Triple therapy also significantly reduced the proportion of PD-1+/CD8+ T cells in the spleens of tumor-bearing mice compared to placebo and compared to D+anti-PD-1 (Figure 4E). However, this effect was not different from F+anti-PD-1 or F alone. F+D showed a higher proportion of splenic PD-1+/CD8+ cells compared to placebo, confirming our in vitro findings on T cells. This increase was abrogated by addition of anti-PD-1 in the triple therapy group. In the macrophage population, only the triple therapy group showed a significant decrease in CD206 positivity in both tumors (Figure 4F) and spleens (Figure 4G) compared to placebo. F+D slightly increased CD206+ macrophages in spleens but it did not reach statistical significance, and also did not change the proportion of M2+ macrophages in tumors, which was considerably elevated in the placebo condition at the time of tumor harvest (97% of total macrophages). The main effect in vivo was induction by F+D of an inflamed TME, with maximal PD-1 expression in both the TME and the splenic compartment, effects that were reversed by anti-PD-1antibody. Due to these effects, lung tumors might best respond to ICB if anti-PD-1 antibody was given after F+D in a sequential approach.

### 2.4. The Triple Therapy Given Sequentially Significantly Improved the Antitumor Effect

**Figure 5:**
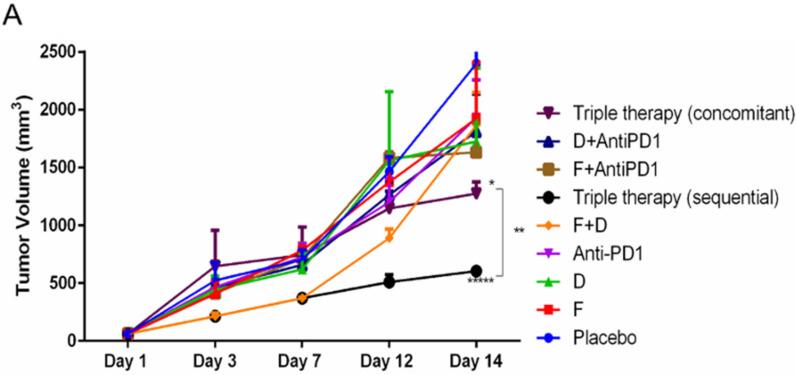

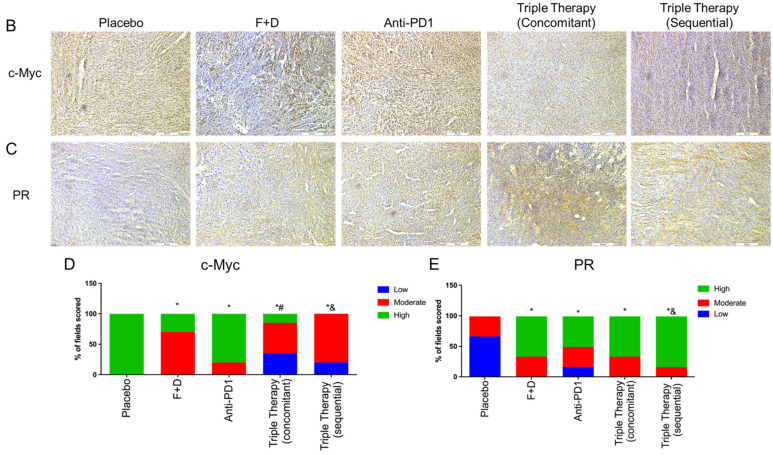
Effect of triple therapy using a concomitant compared to sequential approach on growth of s.c engrafted FVBW-17 cells into syngeneic hosts. (**A**). Mice were randomized to receive placebo, fulvestrant, dacomitinib, anti-PD-1, F+D, F+anti-PD-1, D+anti-PD-1, or triple therapy. Triple therapy in the concomitant group received all three treatments simultaneously. In the triple therapy sequential group, mice were treated from days 1 to 7 with F and D, then switched to anti-PD-1 for days 8 to day 14. Results represent relative mean tumor volume ± SEM of 5 tumors/group. Data was assessed by ANOVA followed by *t*-test and considered significant at *p* ≤ 0.05. Significance compared to placebo is indicated by * *p* < 0.05, significance comparing placebo to sequential treatment indicated by ***** *p* < 0.00005; ** indicates significance comparing concomitant and sequential therapy at *p* < 0.005. (**B**). Representative IHC images of c-Myc expression and (**C**). PR expression in FVBW-17 tumors, following two weeks of placebo, F+D, anti-PD-1, concomitant triple therapy or sequential triple therapy. Scale bar = 50 µm. (**D**,**E**). Quantification of c-Myc (**D**) and PR (**E**) IHC performed on sections from three tumors per group. Data are presented as the proportion of fields that were scored low, moderate, or high. *p* ≤ 0.05 was considered significant as measured by Chi squared and Fisher’s exact tests: * indicates significance compared to placebo, *& indicates significane compared to all other groups, *# indicates significane compared to anti-PD-1, F+D and placebo.

To determine whether immediate effects seen in the TME would translate into better antitumor effects, we assessed the tumor volume over two weeks of treatment, using the FVBW-17 model. FVBW-17 cells are aggressive and progress rapidly, limiting the therapeutic window. One week after cell injection, mice were randomized to receive F, D, anti-PD-1 antibody, dual therapies, and triple therapy (concomitantly or sequentially) for two weeks. In the sequential group, mice were given F+D for 7 days, then switched to anti-PD-1 for another week. Triple therapy provided significantly better growth inhibition than single agents or any of the dual therapy groups, with the sequential approach being the most effective regimen (Figure 5A). F+D showed a strong initial antitumor effect, which deteriorated by the beginning of the second week. However, switching to anti-PD-1 in the second week (sequential group) was able to maintain the antitumor effect and inhibit the tumor outgrowth. The mean tumor volume in the sequential group was four-fold less than placebo (*p* < 0.00005) and two-fold less than concomitant treatment (*p* < 0.005). The concomitant group showed a two-fold decrease in tumor growth compared to placebo (*p* < 0.05). The triple therapy given sequentially but not concomitantly demonstrated synergy, with a combination ratio of 1.35 (>1 indicates strong synergy and <1 less than additive effects). Therapeutic response was also examined as an endpoint, defined as an individual tumor with a smaller volume than the smallest tumor in the placebo group. F+D and F+anti-PD-1 showed a response rate of 60% (3 of 5), while D+anti-PD-1 had the same response rate as anti-PD-1 alone (20% [1 of 5]). For both triple therapy groups, the response rate was 100%.

We also investigated the effect of triple therapy on the protein expression of two of the genes in the ERβ/HER2/HER3 network signature (c-Myc and progesterone receptor (PR)) by IHC. C-Myc is upregulated and PR is downregulated in ERβ+ NSCLC high risk patients [14]. As expected, F+D significantly downregulated c-Myc and upregulated PR compared to placebo. Figure 5B,C shows a representative IHC image for c-Myc and PR, and Figure 5D,E shows quantitative scoring of multiple fields within tumors. Triple therapy given sequentially enhanced F+D effects, particularly by upregulating PR. Collectively, these data suggest that an ICB given after F+D retains or improves the activity of F+D on lung tumor cells.

**Figure 6:**
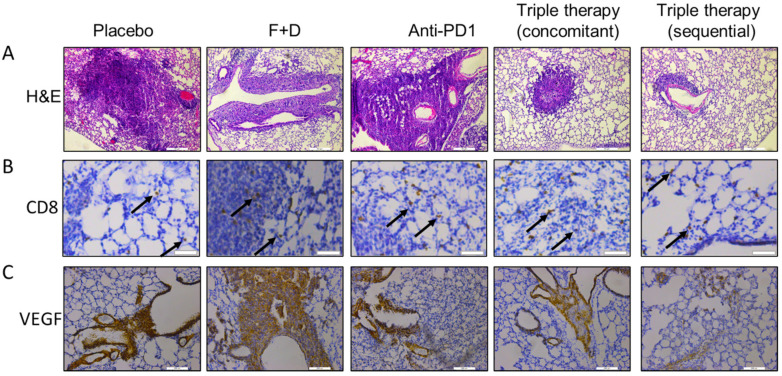

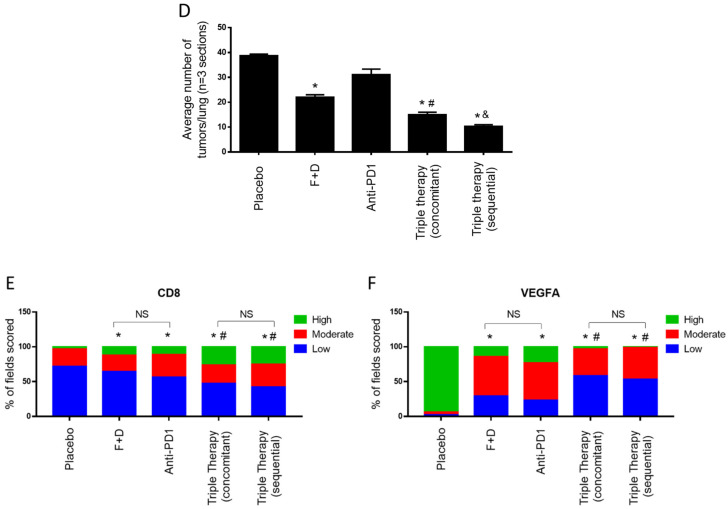
Effect of concomitant compared to sequential triple therapy on lung tumor development of FVBW-17 cells seeded into the lungs via tail vein injection. FVB/N mice were engrafted with FVBW-17 cells by i.v. injection and then treated for two weeks with placebo, F+D, anti-PD-1, concomitant triple therapy or sequential triple therapy with the same dosing schedule as in Figure 5. (**A**). H&E staining of lung sections, showing representative tumors established in the pulmonary microenvironment by treatment group. Scale bar = 200 µm. (**B**). Representative images of lungs stained with anti-CD8; black arrows indicate CD8+ cells. Scale bar = 50 µm. (**C**). Representative images of tumors stained with anti-VEGFA. Scale bar = 200 µm. (**D**). Mean number of lung tumors following two weeks of treatment. Tumors were enumerated in lungs from four mice per group, using five 5-µm sections taken from different predetermined depths. Data were analyzed by ANOVA followed by *t*-test and considered significant at *p* ≤ 0.05: * indicates significance compared to either placebo or anti-PD-1, # indicates significance compared to placebo, F+D, or anti-PD-1, and & indicates significance compared to all other treatment groups including concomitant triple therapy. (**E**). Quantification of CD8 IHC from three lungs per group presented as the proportion of low, moderate, or high staining. (**F**). Quantification of VEGFA IHC from three lungs per group, presented as the proportion of low, moderate, or high staining. *p* ≤ 0.05 was considered significant, as measured by Chi squared and Fisher’s exact tests: * indicates significance compared to placebo group, # is significant compared to either F+D or anti-PD-1.

The antitumor effects of triple therapy were confirmed in a more physiologically-relevant pulmonary model. FVBW-17 cells were injected intravenously by tail vein, where they seeded in the lungs and formed multiple tumors, mainly near the pulmonary blood vessels (Figure 6A, Supplemental Figure S4). After 5 days, mice were randomized to receive placebo, F+D, anti-PD-1, or the triple therapy, either concomitantly or sequentially, as in Figure 5. Lungs were harvested, and 5 slides were cut from predetermined depths in 4 animals per group. Slides were H&E stained and analyzed for the presence of lung tumors (20 slides/group). Sequential triple therapy resulted in significantly fewer lung tumors compared to placebo, F+D, anti-PD-1, or concomitant treatment (Figure 6A,D): the mean number observed was 10 tumors per slide in the sequential group, compared to 15 in the concomitant group, 20 tumors in the F+D group, 31 tumors with anti-PD-1, and 38 tumors with placebo. The combination ratio was 1.4 with concomitant therapy and 2.14 with sequential therapy, indicating strong synergy. F+D showed a significant reduction in lung tumors compared to placebo (*p* < 0.001) or anti-PD-1 (*p* < 0.0005), while anti-PD-1 alone had less tumors but was non-significant (Figure 6D). Tumors from sequential treatment showing a less aggressive growth pattern overall than the other groups (Figure 6A, Supplemental Figure S4). Placebo and anti-PD-1 groups produced lung tumors that grew in thick, dense sheets, with evidence of angiogenesis. With F+D treatment, tumors were less dense with less aggressive edges. With concomitant triple therapy, the tumors showed more uniform growth, and some invasive edges. Sequential treatment showed evidence of involution or breakdown in the tumor architecture. These data confirm that the enhanced antitumor effect of sequential therapy observed in the flank model is recapitulated in the pulmonary microenvironment.

Lungs were analyzed by IHC for two markers related to anti-tumor immunity: CD8+ T cell infiltration and VEGFA. The number of CD8+ T cells was significantly increased in the lungs of triple therapy groups compared to placebo, F+D, or anti-PD-1 (Figure 6B,E); 25% of the fields scored high in both triple therapy groups compared to 3% in placebo, 10% in anti-PD-1, and 12% in F+D groups. VEGFA was markedly reduced with triple therapy (both regimens) compared to placebo, F+D, or anti-PD-1 (Figure 6C,F); 94% of the fields scored high in the placebo group, compared to 23% in anti-PD-1, 14% in F+D, 3% after concomitant triple therapy, and 1% after sequential therapy. This shows that modulation of immunologic endpoints is equally effective with concomitant and sequential treatments, even though drugs in the sequential group were each given for half the time. Timing of therapy may be more important than overall dose, with reduced exposure to ICB when given sequentially providing equivalent effects.

## 3. Discussion

This study demonstrated the immunomodulatory effects of F+D, a treatment that had previously produced anti-tumor synergy in models of ER+ NSCLC in immunocompromised mice [7]. ER (primarily ERβ) is expressed in over 70% of NSCLC, including *KRAS*+ tumors [7,14]. F+D had positive effects on synergy against the growth of murine *KRAS* mutant adenocarcinoma cells, as well as the production of a strong immune cell infiltrate in syngeneic hosts implanted with tumors. The dual treatment had the negative effect of blocking the activity of kinases important for immune cell function. Dual treatment also induced PD-1 in immune cells and increased the number of TAMs in the TME. The therapeutic effects of F+D in a syngeneic host were enhanced with the addition of anti-PD-1 antibody. Triple therapy produced a high CD8+ T cell infiltrate, with lower overall PD-1 expression. The ability of F+D to impair the oncogenic ERβ/HER network was not reduced by addition of anti-PD-1 antibody, and may have been enhanced, especially when anti-PD-1 antibody was given after the targeted treatments. Triple therapy was also most effective against tumor growth, when used sequentially with an ICB following targeted therapy. Sequential therapy was superior to concomitant triple treatment, producing synergy in both s.c. flank tumors and in adenocarcinoma cells colonizing the lungs. Sequential therapy also improved the effects of anti-PD-1 therapy alone, providing a rationale for this sequential regimen to treat NSCLC.

Several factors contribute to the mechanism by which the sequential treatment produced greater efficacy. Dacomitinib, as a pan-HER TKI, also inhibits Syk and Src kinases on macrophages and T cells. Syk and Src kinases are essential for regulating innate and adaptive immune responses; drugs that target these kinases are being investigated as treatments for inflammatory and hematological disorders [24,25]. Dacomitinib impaired the phagocytic function of macrophages and reduced cytokine production in CD8+ T cells, and potently induced PD-1 in both cell types. Anti-PD-1 antibodies are known to reprogram the TME toward a more immune supportive milieu [26,27]. ICBs also promote an M1-like phenotype and restore the phagocytic functions of PD-1+ macrophages [5]. However, to be effective clinically, these inhibitors require an inflamed TME and high expression of PD-1/PD-L1 pathways [28]. Sequential treatment allows F+D to induce an inflamed TME, while tumor cells are simultaneously being inhibited by repression of the ER/HER oncogenic network. The delayed administration of anti-PD-1 antibody, in the absence of a targeted therapy that can suppress kinases important for immune cell activity, is then able to maximize the effectiveness of an ICB. Sequential treatment produced the best reduction in tumor volumes, and the remaining tumor cells showed a less aggressive gene signature. 

The syngeneic FVBW-17 lung tumor cells used for these studies contain both a G12D *KRAS* activating mutation and a *TP53* inactivating mutation, and have the genetic signature of a classic alkylating agent, with a high tumor mutation burden [11]. *KRAS* mutant lung cancers are highly dependent on the HER network, especially HER2 and HER3 [12,13], and *KRAS* mutant lung cancer patients harboring *TP53* co-mutations were shown to have the best objective response rate to PD-1 blockade compared to those with *STK11/LKB1* co-mutations or *KRAS* only mutations in the CheckMate-057 trial [29]. We previously reported enhanced blockade of the HER2/HER3 network with F+D by inhibition of ER/HER crosstalk in lung cancer, and we showed that F+D was synergistic in A549 cells, a human *KRAS* mutant model [7]. The findings reported here extend these observations to the immunocompetent setting. FVBW-17 cells showed a partial response to the anti-PD-1 antibody as a single therapy. Whether the triple therapy we tested would be highly effective in immunocompetent models of lung cancer with other genetic drivers remains to be determined. *EGFR* wild-type lung cancer, without evidence of other drivers, also responded to F+D previously [7], indicating that triple sequential therapy might be effective when the oncogenic driver is unknown. Anti-PD-1 antibody was chosen because PD-1 was strongly induced by the targeted therapies used. Whether the findings extend to other types of ICBs remains to be determined.

This triple sequential regimen might also be an option for *EGFR* mutant NSCLC, since D is approved for the treatment of *EGFR* mutant lung cancer [30]. We previously showed that F+D produced synergy in *EGFR* mutant lung cancer cells, with enhanced blockade of the HER2/HER3 network [7]. To date, ICB therapy in *EGFR* mutant lung cancer has been disappointing. *EGFR* mutant tumors are generally considered to have low immunogenicity, unlike *KRAS* mutant tumors that tend to be highly immunogenic [31]. Concomitant use of an EGFR inhibitor with an ICB might not be effective, as the tumor lacks immune infiltration and requires time to develop an immune response. Potential negative effects on immune kinases could explain the moderate activities of EGFR TKIs in *EGFR* wild type or *KRAS* mutant models. In fact, anti-PD-1/PD-L1 agents are approved for *EGFR* mutant NSCLC, only after progression on EGFR TKIs, suggesting EGFR TKIs favorably modulate the TME with regard to anti-PD-1/PD-L1 efficacy [32]. Extensive cancer cell death following EGFR TKI treatment in *EGFR* mutant NSCLC models induces a high immune cell infiltration that includes dendritic cells, macrophages, and cytotoxic CD8+ T cells [33]. Clinically, patients who relapsed on first-line EGFR TKIs experienced a change in the TME that tended to be highly immunosuppressive [32]. These are all indications that sequential ICB might be more effective than a concomitant approach when combining an ICB with targeted therapy. Whether F could be combined with other EGFR-TKIs such as osimertinib to achieve synergy when used with sequential ICB for *EGFR*-mutant lung cancer has also not been studied. However, osimertinib has less effects on HER2/HER3 signaling than dacomitinib, so it might not block the ER/HER network effectively, an effect we previously found important in synergy [7].

Treatment of *KRAS* mutant lung cancer remains a clinical challenge, despite the recent approval of the first KRAS targeted inhibitor sotorasib for *KRAS* G12C mutant NSCLC based on the promising activity in the CodeBreak100 trial [34]. Several other KRAS G12C inhibitors are currently being tested in clinical trials, including adagrasib (NCT04685135; NCT04975256; NCT04613596; NCT03785249), JNJ-74699157 (NCT04006301) and GDC-6036 (NCT04449874). However, the current first line standard of care still consists of immunotherapy with or without platinum-based chemotherapy. Sotorasib is not effective against the two most common *KRAS* mutations, G12D and G12V, and resistance develops at both upstream (EGFR, HER2, FGFR) and downstream (MEK) pathways. The results of several clinical studies combining a pan-EGFR TKI with MEK inhibitors in *KRAS* mutant NSCLC have also reported limited therapeutic benefit [35,36,37]. Timing and treatment sequence may be critical for combination treatment approaches, especially for immunotherapy, since concomitant administration of an ICB with targeted therapy is associated with greater toxicity. In this regard, several studies showed a lack of improvement, with a significant increase in side effects, that precluded further evaluation of concomitant administration of EGFR-TKIs with an ICB [38,39,40,41]. Toxicity may be lower when ICB is given after targeted therapy [42]. Sequential ICBs administered after well-chosen targeted therapy, which limits overall drug exposure and may prevent toxic drug interactions, may reduce unacceptable side effects, while increasing the overall therapeutic effect. In support of this, the Src inhibitor dasatinib was recently proposed as a pharmacological on/off switch to control CAR T cell activity transiently and reduce its lethal toxicities [43]. Additionally, a sequential versus combinatorial treatment regimen of anti-PD-1/PD-L1 lead-in before a MAPK inhibitor was shown to maximize antitumor immunity and efficacy in murine models of melanoma, colorectal, and pancreatic cancer [44]. Clinically, fulvestrant was well tolerated when previously combined with the first-generation EGFR TKIs gefitinib [45] and erlotinib [46]. While we did not observe any signs of toxicity with sequential triple therapy, future studies should focus on confirming the long-term safety of this treatment and assess the benefit in mice, using survival as the endpoint. Future studies should also assess the immune response in depth during sequential treatment, by measuring more thoroughly the activation and exhaustion states of different immune cell subsets, including M1 and M2 macrophages and T cell subsets, as well as the interplay of the TME with the systemic immune system. 

## 4. Materials and Methods

### 4.1. Chemical Reagents

Dacomitinib (Selleckchem, Houston, TX, USA) and fulvestrant (Tocris, Minneapolis, MN, USA) were dissolved in DMSO. DMSO, as a control, was given at the same amount as the maximum DMSO used in treatment groups. For in vivo studies, peanut oil was used to dilute the dose of fulvestrant for s.c. administration. Dacomitinib was further dissolved in polyethylene glycol (30%) and PBS for oral gavage administration. Rat monoclonal blocking anti-mouse PD-1 (clone RMP1.14) and isotype control (rat IgG2a, clone 2A3) were purchased from BioXcell and prepared in InvivoPure dilution buffer (BioXcell, Lebanon, NH, USA). Mouse IFN-ϒ, mouse TNF-α, and mouse VEGF ELISA kits were purchased from R&D Systems (Minneapolis, MN, USA). 

### 4.2. Cell Viability Assay

FVBW-17 cells were grown in DMEM medium supplemented with 10% fetal bovine serum (FBS), 1X penicillin/streptomycin (Thermo Fisher, Waltham, MA, USA), and 1X GlutaMax (Life Technologies, Carlsbad, CA, USA). A series of dose response experiments were conducted for F and D, to assess the anti-cancer activity using CellTiter 96 reagent (Promega, Madison, WI, USA), as described previously [7]. The CI to evaluate synergy was calculated using the Chou–Talalay method [18] and CompuSyn software. CI < 1 indicates synergism, CI = 1 indicates an additive effect, and CI > 1 indicates antagonism. 

### 4.3. Isolation and Culture of Murine BMDMs

Femurs and tibia bones were obtained from an FVB/N mouse and rinsed with ethanol then with DMEM medium. The ends of each bone were cut and flushed with DMEM medium and collected in a 50 mL tube. A single cell suspension was made by drawing media up and down using a syringe. Cells were centrifuged for 5 min at 4 °C. The cell pellet was resuspended in ACK lysis buffer (4.15 g NH_4_Cl, 0.5 g KHCO_3_, 0.1 mM EDTA), centrifuged at 1200 rpm for 5 min, and the pellet was resuspended in DMEM supplemented with 10% FBS, 1X penicillin/streptomycin, and 1X Glutamax. 10 × 10⁶ cells were plated in 15 cm plates in a final volume of 30 mL. Half of the culture medium was replaced with fresh DMEM on the day 4. Experimental treatments began on day 6.

### 4.4. Harvesting and Activation of Mouse CD8+ T Cells

CD8+ T cells were isolated from FVB/N mouse spleen using a CD8+ T cell isolation kit (Miltenyi Biotec, Auburn, CA, USA). Spleen was minced and pressed through a strainer and washed with PBS. Collected cells were centrifuged for 10 min at 300× *g*. The pellet was resuspended in ACK lysis buffer (2 mL) for 10 min at 4 °C. Lysis was terminated with 30 mL PBS, and the suspension was then centrifuged for 10 min at 300× *g*. The pellet was resuspended with culture media (RPMI + 10% FBS). CD8+ T cells were isolated using magnetic cell sorting by negative selection using a CD8+ T cell isolation kit (Miltenyi Biotec, Auburn, CA, USA). CD8+ T cells were activated using Dynabeads Mouse T-Activator CD3/CD28 beads (Thermo Fisher) using RPMI + 10% FBS for 24 h. Recombinant mouse IL-2 (30 U/mL) was added, according to the manufacturer’s protocol for T cell expansion. Activation status was confirmed by measuring IFN-ϒ and TNF-α release by ELISA.

### 4.5. Immunoblotting

Western blotting experiments were conducted as described previously [7]. Primary antibodies are listed in Supplemental Table S1.

### 4.6. ELISA Assays

CM from treated cells were collected and used without concentration for ELISA. Cell quantification was performed for normalization for each sample. ELISA was performed according to the manufacturer’s protocol.

### 4.7. Macrophage Phagocytic Activity

BMDMs were cultured at a density of 8000 cells/well in 8-chamber slides. The next day, BMDMs were treated with DMSO, F, D, or F+D for 6 h. Phagocytic activity was assessed using a Phagocytosis Assay Kit (IgG FITC; Cayman Chemical, Ann Arbor, MI, USA) according to the manufacturer’s protocol. Briefly, after treatment, latex beads (FITC complex) were added to each group at a final dilution of 1:250 and incubated with the cells for 2 h at 37 °C. Cells were washed and counterstained with Hoechst 33342 (40 μM) for 10 min. After two washes, BMDMs were visualized at 40X magnification with a Leica DM 4000B LED microscope using LAS4.7 software (Allendale, NJ, USA).

### 4.8. Real Time Quantitative Polymerase Chain Reaction (RT-qPCR)

RT-qPCR experiments were conducted as described previously [7]. Glyceraldehyde 3-phosphate dehydrogenase (*GAPDH*) was the internal control. The primer sequences are listed in Supplemental Table S2.

### 4.9. Flow Cytometry Analysis

After treatment, macrophages and CD8+ T cells were collected in FACS tubes at 1 × 10⁶ in 100 µL of PBS. Fc blocking antibody was added at 1 µL/one million cells for 15 min at 4 °C. Cells were washed with FACS buffer (PBS + 2%FBS and 5 mM EDTA) and stained for 30 min with viability dye (eFluor 780), anti-F4/80 BV785, anti-PD-1 FITC, and anti-CD8 APC. After incubation, cells were washed with FACS buffer and resuspended in FACS buffer supplemented with 0.5% paraformaldehyde at a final volume of 0.5 mL. Cells were analyzed using LSR Fortessa (BD Biosciences, Franklin Lakes, NJ, USA) and FACsDiva software (BD Biosciences, Franklin Lakes, NJ, USA).

For tumor and spleen flow cytometric analysis, Ficoll-Paque isolation media (Thermo Fisher) was used to separate viable mononuclear cells including lymphocytes and monocytes, as described [47]. Briefly, tumors and spleens were collected from three mice per group. Tumors were mechanically dissociated using razor blades and incubated with 1× collagenase (Sigma Aldrich) and 1× DNase I (Invitrogen, Waltham, MA, USA) for 30 min at 37 °C 5% CO_2_, with periodic mixing. Spleens were only incubated with a digestive enzyme buffer for 10 min. Tissues were filtered through a strainer and washed with RPMI + 10% FBS. Cell counts were performed, and samples were separated into 2 FACS tubes with equal volume of FACS buffer (T cells and myeloid cells panels). Fc blocking was performed as above, and samples were stained with two panels: T cell panel (CD45/CD3/CD8/PD-1) and myeloid cell panel (CD45/CD11b/F480/CD206). Antibodies used are listed in Supplemental Table S3. Fluorescence Minus One (FMO) controls were used for data interpretation. Debris and dead cells were excluded by side scatter vs. forward scatter (SSC vs. FSS).

### 4.10. In Vivo Animal Studies

For the syngeneic s.c. model, female FVB/N mice (4- to 6-weeks old) were injected in one flank with 0.5 × 10⁶ FVBW-17 cells (100 µL in PBS). After one week, when tumors were palpable, mice were randomized into 5 mice/group and received the following treatments: placebo (DMSO + isotype IgG control (250 µg intraperitoneal (i.p) twice a week); F (30 mg/kg s.c. twice a week); D (10 mg/kg daily through oral gavage); anti-PD-1 (250 µg i.p. twice a week); F+D; F+anti-PD-1; D+anti-PD-1; or triple therapy either concomitantly or sequentially (F+D were given for one week then switched to anti-PD-1 alone for another week). Tumors were measured using calipers, using the formula: volume = (length × width^2^)/2. For the intrapulmonary colonization model, mice were injected with 1 × 10⁶ FVBW-17 cells through the tail vein. After 5 days, mice were randomized into 8 mice/group to receive placebo (DMSO +isotype IgG control (250 µg i.p. dose twice a week)), F+D (F at 30 mg/kg s.c. twice a week and D at 10 mg/kg daily by oral gavage), anti-PD-1 (250 µg i.p. twice a week), or triple therapy, either concomitantly or sequentially. Mice were treated for two weeks. All experiments were approved by the University of Minnesota Institutional Animal Care and Use Committee. Synergy for drug combination in vivo was assessed by the combination ratio method [48], as previously used by us [7]. A combination ratio >1 indicates synergy while a ratio <1 indicates antagonism.

### 4.11. Immunohistochemistry (IHC) 

Tissues were prepared for IHC, as described previously [7]. Tissue sections used for the analysis were blindly scored as low (<30% of the fields stained positive), moderate (30–60% of the fields stained positive), or high (>60% of the fields stained positive). Then, 20 fields were examined per slide (60 fields total per treatment group). Data were expressed as the percentage of fields examined that scored low, moderate, or high. See Supplemental Table S1 for primary antibodies.

### 4.12. Cytometric Bead Array

Cytokine production was measured in conditioned media from 125,000 FVBW-17 cells cultured in 500 µL using cytometric bead arrays (CBA; BD Bioscience) including the mouse inflammation kit measuring IL-6, IL-10, CCL2, IFNγ, TNFα, and IL-12p70, CBA Flex sets for IL-4 and IL-1β, and CBA TGFβ kit per the manufacturer’s instructions. Data and was collected on a 4-laser BD LSR Fortessa flow cytometer and analyzed with FlowJo V10.7 (BD Biosciences).

### 4.13. Statistical Analysis

Data were reported as mean ± standard deviation (SD) or standard error of the mean (SEM). Significance, determined by AVOVA followed by two-tailed Student’s *t* test of individual group comparisons, was set at *p* ≤ 0.05. IHC experiments were analyzed for statistical significance by Chi-square and Fisher’s exact tests to compare the frequency of low, moderate, and high staining scores. Cell culture experiments were conducted at least twice with at least three replicates. Flow cytometry was run twice using a pool of 5 tumors per group. 

## 5. Conclusions

This study provides new insight into a novel treatment approach, which combines targeted agents with an ICB, to treat highly-aggressive *KRAS* and *TP53* mutant lung cancer; a subset of NSCLC patients without effective first-line therapies. The potential of combining F and D with anti-PD-1 in a sequential approach is supported by the ability of F+D to induce potent cancer cell death, downregulate the ER/HER gene expression network in tumor cells, enhance immune cell infiltration, and promote a relatively inflamed TME with increased PD-1 expression. Many of these features should contribute to the efficacy of anti-PD-1 therapy to prolong treatment benefits. Future studies should focus on exploring the immune cell context in depth during triple therapy, to determine the contribution of immune cell exhaustion during F+D therapy to the superiority of sequential treatment. The data presented also highlight the importance of understanding the modulatory effects of targeted therapy on immune cells and the TME, in addition to effects on tumor cells and the importance of the timing of PD-l blockade in combinatorial approaches. This is crucial for designing therapeutic regimens with improved efficacy and safety.

## Data Availability

The data presented in this manuscript form part of the doctoral dissertation of Abdulaziz A. Almotlak, in partial fulfillment of the Doctor of Philosophy degree in Pharmacology at the University of Minnesota. The dissertation is available through the University of Minnesota Library system.

## Supplementary Materials

The following are available online at https://www.mdpi.com/article/10.3390/ijms23010081/s1.
