# Supplementary material for: Targeting the ERβ/HER Oncogenic Network in KRAS Mutant Lung Cancer Modulates the Tumor Microenvironment and Is Synergistic with Sequential Immunotherapy
Source: Int J Mol Sci. 2021 Dec 22;23(1):81. doi: 10.3390/ijms23010081 (PMC8745184; doi:10.3390/ijms23010081)
Supplement: Supplementary file 1 [file ijms-23-00081-s001.zip › ijms-1422250-supplementary.pdf]

Supplemental Figure S1. FVBW-17 murine cancer cells express EGFR, HER2, HER3 ERβ and PD-L1. FVBW-17 whole cell lysates were immunoblotted with the indicated antibodies as described in Methods.

Supplemental Figure S2. Resting state BMDMs were plated on 96 well plates with 5,000 cells per well. The following day, fulvestrant (5μM) and/or dacomitinib (10μM) were added and incubated with the cells for 6hr, 24hr or 48hr at 37°C in humidified CO_2 incubator. Cell viability was determined using the Cell Titer Aqueous One Solution MTS assay (Promega, Madison, WI). Control values were set to 100 for relative cell proliferation. Cell Titer reagent (20μl) was added to each well and plates were incubated for 1 hour. Results are the mean ± S.D. with 6 samples per experimental treatment.

Supplemental Figure S3. The gating strategy used to evaluate the CD45+/CD3+/CD8+/PD1+ and CD45+/CD11b+/F480+/CD206+ populations in the placebo control group is depicted.

Supplemental Figure S4. H&E staining of lungs from FVB/N mice engrafted with FVBW-17 cancer cells by tail vein injection. Representative sections of lungs from different treatment groups illustrate the histology of tumors detected. Lungs harvested after 2 weeks of therapy were subjected to H&E staining as described in Methods.

Supplemental Table S1. Primary antibodies used for western blotting and IHC.

Antibody	Dilution	Manufacture

p-EGFR (Tyr1068)	1:1000	Cell signaling Technology
p-HER2 (Tyr1221/1222)	1:1000	Cell signaling Technology
p-HER3 (Tyr1289)	1:1000	Cell signaling Technology
p-Akt (Ser473)	1:1000	Cell signaling Technology
p-MAPK (Tyr202/204)	1:1000	Cell signaling Technology
p-Syk (Tyr525/526)	1:1000	Cell signaling Technology
p-Src Family (Tyr416)	1:1000	Cell signaling Technology
ERβ antibody (PAI-310B)	1:1000	Invitrogen
PD-L1 (Ab213480)	1:1000	Abcam
c-Myc	1:1000	Abcam
PR	1:1000	Abcam
VEGFA	1:200	Abcam
Mouse CD8	1:1500	Abcam

Supplemental Table S2. Primer sequences for RT-qPCR analysis.

**Primer**	**Sequence**
GAPDH	forward ’GGAGCGAGATCCCTCCAAAAT-3’<br>reverse 5’GGCTGTTGTCATACTTCTCATGG-3’
IL-10	forward 5’-CTG GAC AAC ATA CTG CTA ACC G-3,<br>reverse 5-GGGCATCACTTCTAC-3’
CD206	forward 5’CAGGTGTGGGCTCAGGTAGT 3’,<br>reverse 5’TGTGGTGAGCTGAAAGGTGA 3’
Pdcd1	forward 5’- ATGTGGGTCCGGCAGGTACC-3’,<br>reverse 5’TCAAAGAGGCCAAGAACAATGTC3’

Supplemental Table S3. Antibodies used for flow cytometry analysis.

**Antibody**	**Manufacture**
Anti-mouse CD45 APC-eFluor 780	eBiosciences
Anti-mouse CD3e PerCP-Cy5.5	BD Biosciences
Anti-mouse CD8a Super Bright 600	eBiosciences

Anti-mouse CD279 (PD1) FITC	Biolegend
Anti-mouse CD45 APC-eFluor 780	eBiosciences
Anti-mouse CD11b APC	BD Biosciences
Anti-mouse F480 Alexa Fluor 700	Thermo Fisher
Anti-mouse CD206 PE	Biolegend

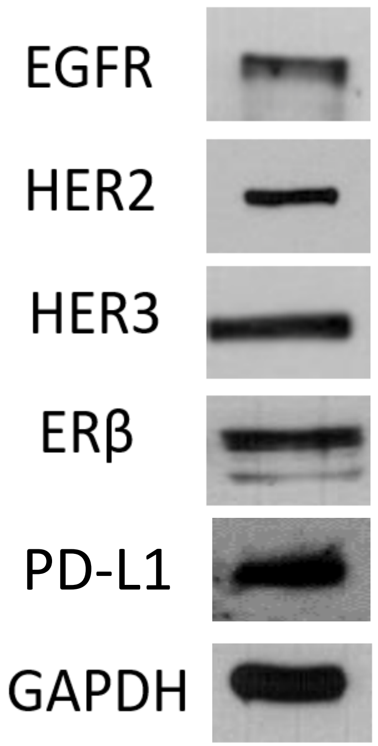

Supl Figure S1

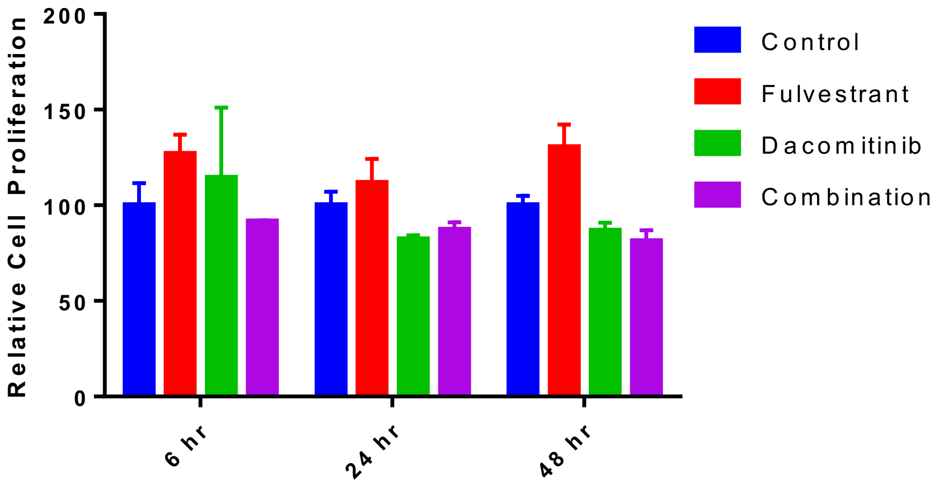

Supplemental Figure S2

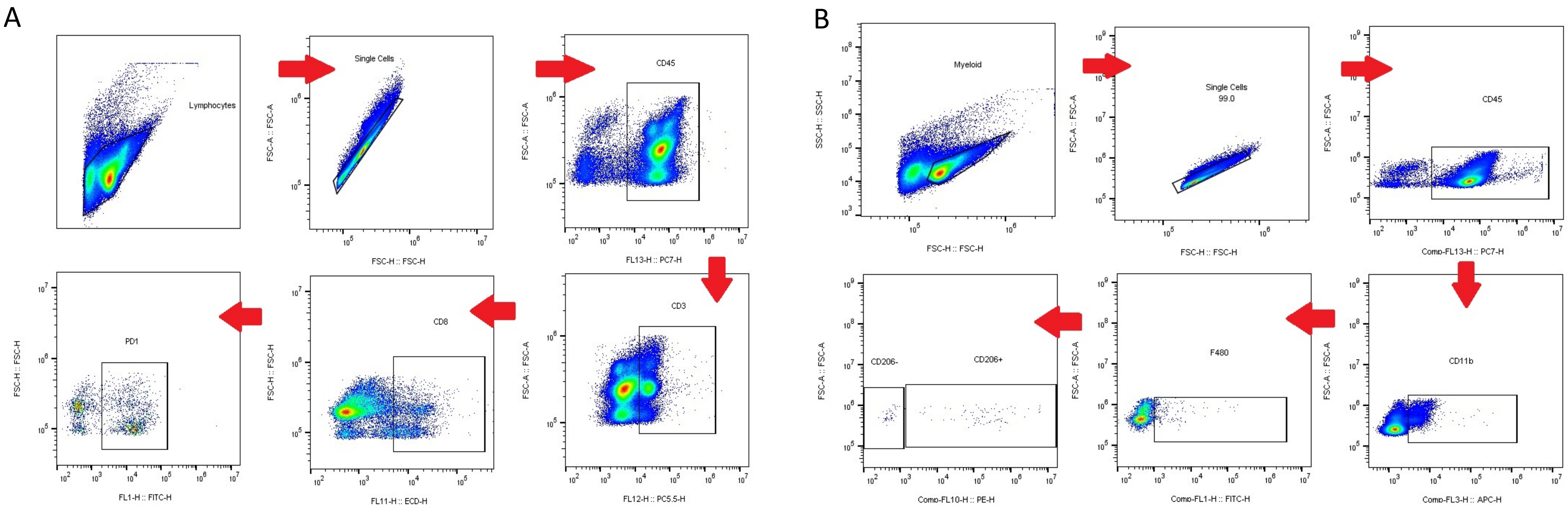

Supl Figure S3

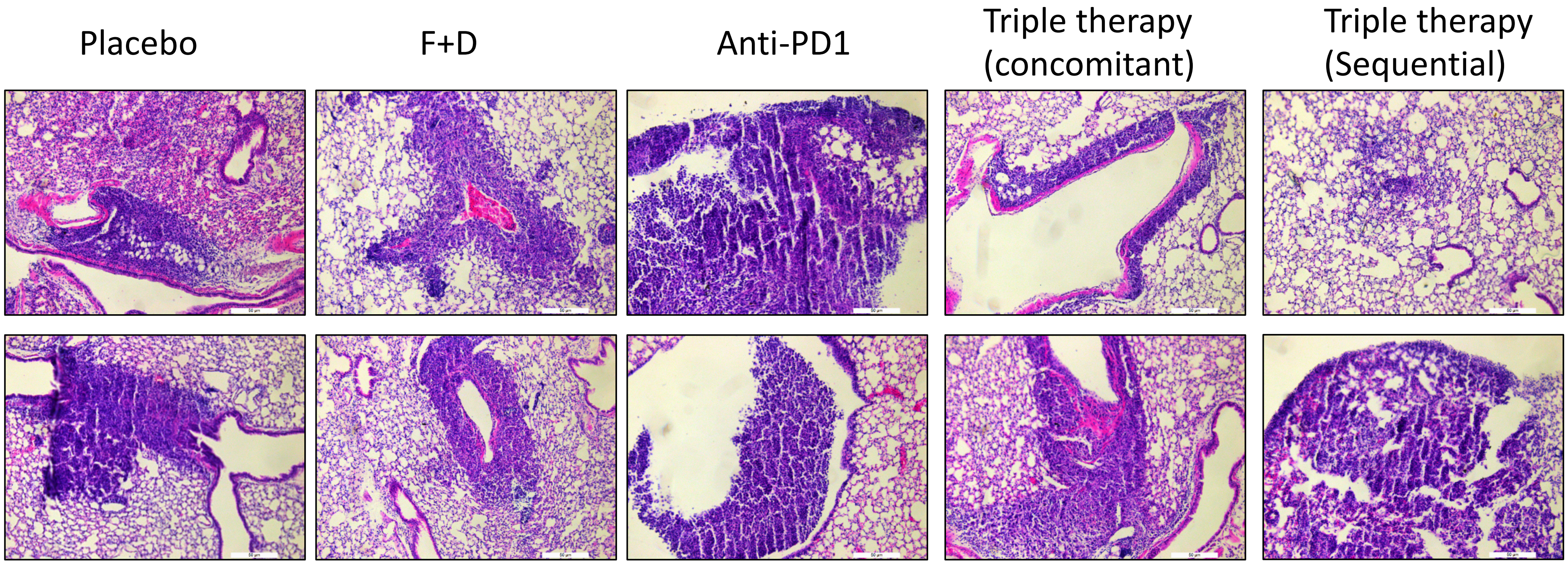

Supl Figure S4
